# The Optimum Mixed Cropping Ratio of Oat and Alfalfa Enhanced Plant Growth, Forage Yield, and Forage Quality in Saline Soil

**DOI:** 10.3390/plants13213103

**Published:** 2024-11-04

**Authors:** Guanglong Zhu, Jiao Liu, Hao Wu, Yiming Zhu, Nimir Eltyb Ahmed Nimir, Guisheng Zhou

**Affiliations:** 1 Joint International Research Laboratory of Agriculture and Agri-Product Safety, The Ministry of Education of China, Yangzhou University, Yangzhou 225009, China; zhuguang2007@163.com; 2 Jiangsu Provincial Key Lab of Crop Genetics and Physiology, Yangzhou University, Yangzhou 225009, China; jiaoliu0407@163.com (J.L.); w13964483386@163.com (H.W.); mx120220721@stu.yzu.edu.cn (Y.Z.); 3 Jiangsu Co-Innovation Center for Modern Production Technology of Grain Crops, Yangzhou University, Yangzhou 225009, China; 4 Faculty of Agriculture, University of Khartoum, Khartoum 11115, Sudan; nimir1000@gmail.com

**Keywords:** saline-alkali land, mixed cropping, growth traits, forage yield, forage quality

## Abstract

The forage shortage is more aggravating than ever before, with husbandry development accelerating and meat and dairy product demand increasing. Salinized soils are important reserve land encouraged to be used for forage production in China. However, the salt-tolerant cultivation techniques for forage crops are still inadequate. Therefore, a field experiment was conducted to study the effects of the mixed cropping ratio of oat and alfalfa on plant growth and physiological traits, forage yield, and forage quality in saline soils. Oat (*Avena sativa* L.) variety of Canadian Monopoly and alfalfa variety of WL525HQ were used, and five mixed cropping ratios (T1 = 100% oat + 0% alfalfa, CK, T2 = 75% oat + 25% alfalfa, T3 = 50% oat + 50% alfalfa, T4 = 25% oat + 75% alfalfa, and T5 = 0% oat + 100% alfalfa) were evaluated. The results showed that plant height, chlorophyll, soluble sugar, starch, antioxidant enzymes, and crude fat were increased firstly and then decreased prominently with decreased oats and increased alfalfa sowing rate; the maximum values showed under T2 but the minimum value under T5 at evaluated growth periods. On the contrary, malondialdehyde and acid detergent fiber were significantly decreased and then increased; the lowest contents were recorded under T2 and highest under T5. Furthermore, the relative growth rate, forage yield, neutral detergent fiber, and crude ash were decreased prominently with decreased oats and increased alfalfa sowing rate, and the highest and lowest values showed under T1 and T5, respectively. Oppositely, the contents of sucrose, proline, N, P, K, relative feeding value, and crude protein were all increased, with the highest contents generated under T2 and the lowest under T1. On the whole, the mixed cropping treatment of T2 showed the best performance in improving both biomass yield and forage quality by enhanced antioxidant enzyme activity, osmotic regulatory substances, and nutrient uptake and utilization. Therefore, this study indicates that 75% oat mixed cropping with 25% alfalfa can be recommended as a salt-tolerant cultivation technique for forage high-yield and high-quality production in moderately saline soil.

## 1. Introduction

According to the statistics, approximately 1 billion ha of land is now affected by salinity worldwide, nearly accounting for 10% of the total land area, which poses a severe threat to the global sustainable development of agriculture [1]. In China, about 100 million hectares of land are affected by different degrees of salinization, over half of which can be used to develop crop production [2]. The development and utilization of saline soils have received more and more attention because cultivated areas are shrinking more quickly than ever due to environmental degradation and urbanization. Under these circumstances, saline-alkali land has become an important reserve land resource, having great development potential. In recent years, the development and utilization of saline lands for agricultural production has become a national strategy to ensure food security in China [3].

In the process of developing and utilizing saline lands, planting forage crops is an important approach to improving saline soil. Planting forage crops can not only increase soil organic matter and improve soil structure but also solve the shortage of forage for livestock, thus increasing farmers’ income. Oat (*Avena sativa* L.) and alfalfa (*Medicago sativa* L.) are the main forage crops for livestock. Feeding with high-quality forage of oats and alfalfa can significantly increase milk yield and quality rather than feeding with straw and fodder concentrate [4]. Oat grain is rich in protein, fat, fiber, and minerals, with protein content ranging from 15% to 20% and fat content from 5% to 10%, which are apparently higher than other cereal crops [5]. More importantly, oat is one of the high-quality forages, which are rich in nutrition, juicy and tender, and with good palatability conducive to digestion and absorption by livestock [6]. Alfalfa is famous as a high-protein forage, with 4% crude protein content in fresh grass and 18% in dry grass, as well as rich in calcium, phosphorus, various minerals, and vitamins. These features make alfalfa become the largest cultivated perennial forage crop globally [7]. Both oats and alfalfa have the advantages of high yield, strong resistance, high nutritional value, and palatability to cattle and sheep, meeting the standard of high-quality forage [8,9]. Currently, the forage of oats and alfalfa is in demand largely over supply in domestic; more than 2/3 of alfalfa is relying on imports, which is increasing the cost of animal husbandry [10,11,12]. Therefore, planting oats and alfalfa in saline land is an effective approach to improving saline soil and alleviating the forage shortage.

Saline soils are characterized by high levels of soluble salts, exchangeable sodium (Na^+^), and chloride (Cl^−^), as well as low organic matter content and poor soil fertility, which typically cause stress and damage to plants [13]. Research has shown that single crops under monoculture in saline land suffered various stresses and a lack of competitive advantage, which resulted in growth difficulties and low yield [14]. Mixed cropping is an effective approach to enhancing plant tolerance to stress in saline soil. It has been demonstrated that the mixed cropping system can fully utilize natural resources and integrate the biological advantages of each single species, enhance crop productivity, and improve forage quality. It was reported that mixed cropping grass with legumes could effectively improve the crop canopy structure, increase photosynthetic area, enhance soil nutrient supply, reduce direct competition for soil nutrients by underground roots, increase resource utilization efficiency, and increase yield production in a mixed cropping system [15,16]. Few studies showed that oats and alfalfa can effectively utilize resources such as light, heat, water, carbon dioxide, etc., as well as the root nodules of alfalfa can provide nitrogen to oats, significantly enhancing productivity and forage quality [17].

In the mixed cropping system, the mixed sowing ratio of each single crop is critical for plant growth, productivity, and quality. An inappropriately mixed sowing ratio in a mixed cropping system could inhibit plant growth and decrease productivity. However, little attention has been given to the mixed sowing ratio of oats and alfalfa in mixed cropping systems to alleviate salinity stress to crops in saline soil. We hypothesized that an appropriate combination of oat and alfalfa in a mixed cropping system in saline soil could effectively improve growth, forage yield, and quality through bettering physiological mechanisms. Therefore, a field study was conducted with five mixed ratios of oat and alfalfa to (1) evaluate the effects of different mixed cropping ratios on growth, biomass yield, and forage quality of oat and alfalfa in the saline soils; (2) explore the associated physiological mechanism of the mixed cropping treatments effects on growth of oat and alfalfa; and (3) select the applicable mixed rate for promoting oat and alfalfa production in saline soils.

## 2. Results

### 2.1. Growth Characteristics and Forage Yield

#### 2.1.1. Plant Height

**Figure 1:**
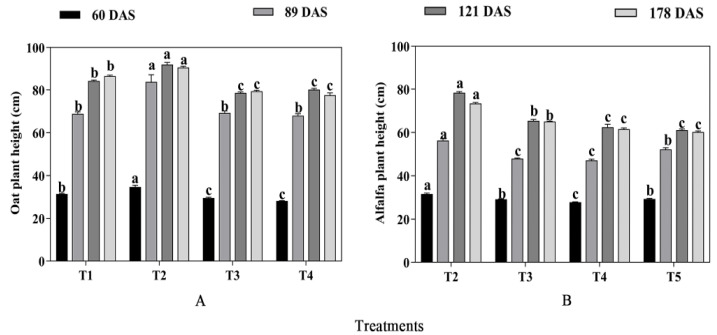
The effect of mixed cropping ratios of oat and alfalfa on plant height in saline soil. Different letters indicate significant differences between different treatments at the same growth stage at the *p* < 0.05 level. (**A**), oat plant height; (**B**), alfalfa plant height.

The mixed cropping significantly affected the plant height of oats and alfalfa (*p* < 0.05, Figure 1). With increased alfalfa and decreased oats mixture sowing rate, the plant height of oats significantly increased initially and then decreased at all growth stages. The tallest oats were observed under T2 mixed cropping treatment (75% oats + 25% alfalfa), which increased plant height of oats by 6.38%, 21.63%, 9.34%, and 4.86% at each growth period compared with T1 treatment, respectively (Figure 1A). In general, the plant height of alfalfa decreased gradually with decreased oats and increased alfalfa sowing rate in the mixed cropping system. The tallest alfalfa plants were also found in the T2 treatment (75% oats + 25% alfalfa) in 60, 89, 121, and 189 DAS, which showed a decrease by −7.86%, 7.80%, 28.33%, and 21.93% compared with the T5 treatment, respectively (Figure 1B).

#### 2.1.2. Relative Growth Rate (RGR)

**Figure 2:**
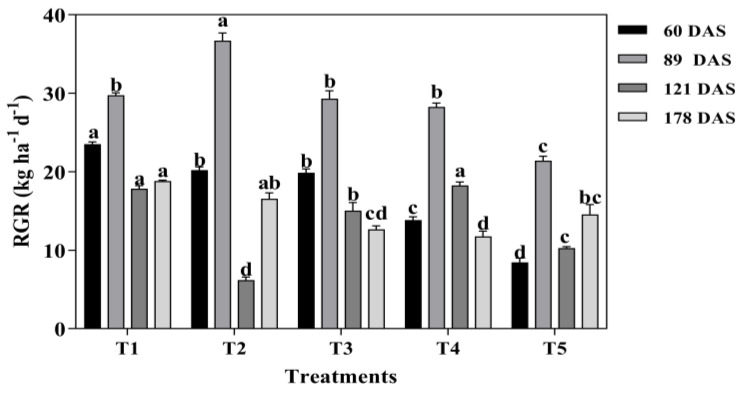
The effect of mixed cropping ratios of oat and alfalfa on relative growth rate in saline soil. Different letters indicate significant differences between different treatments at the same growth stage at the *p* < 0.05 level.

Relative growth rate was significantly affected by mixed cropping treatment (*p* < 0.05, Figure 2). At 60 DAS, the RGR was significantly decreased with decreased oats and increased alfalfa sowing rate. The highest RGR was generated under T1 (except T2 at 89 DAS), which showed 14.13%, 15.50%, 41.19%, and 64.09% higher than T2, T3, T4, and T5, respectively. At 89 DAS, the RGR significantly increased initially and then decreased with increased alfalfa and decreased oats mixture sowing rate. The maximum RGR was observed under T2 mixed cropping treatment, which showed 18.95%, 20.13%, 23.02%, and 71.41% higher RGR than T1, T3, T4, and T5 treatment, respectively (Figure 2). However, the highest RGR showed under T1 and T4 treatments at 121 DAS, but the lowest RGR was under T2 treatment. At 178 DAS, the RGR was significantly decreased with decreased oats and increased alfalfa sowing rate except under T5 treatment. T1 produced the maximum RGR, which was 11.97%, 32.74%, 37.43%, and 22.65% higher than T2, T3, T4, and T5, respectively (Figure 2).

#### 2.1.3. Forage Yield (Fresh Weight and Dry Weight)

**Figure 3:**
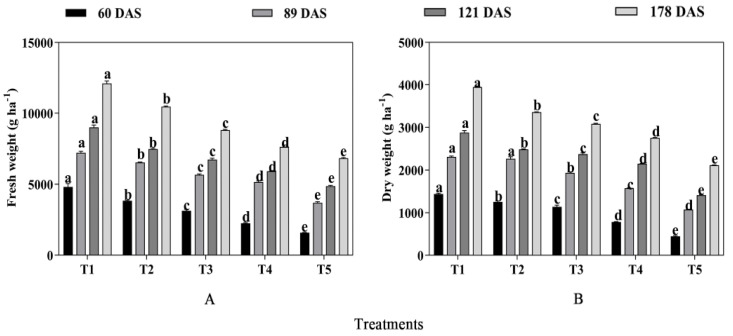
The effect of mixed cropping ratios of oat and alfalfa on fresh weight and dry weight. Different letters indicate significant differences between different treatments at the same growth stage at the *p* < 0.05 level. (**A**), fresh forage yield; (**B**), dry forage yield.

The forage yield of fresh weight and dry weight of oats and alfalfa were significantly affected by mixed cropping treatment (*p* < 0.05, Figure 3). With decreased oats and increased alfalfa sowing rate, the fresh weight and dry weight significantly declined at different growth periods (*p* < 0.05). It is worth noting that the highest forage yield of both fresh weight and dry weight was shown under T1 treatment at each growth stage. Compared with T1 treatment, the forage yield of fresh weight decreased by 20.16% at 60 DAS, 9.37% at 89 DAS, 17.01% at 121 DAS, and 13.54% at 178 DAS under T2 treatment; decreased by 34.82%, 21.44%, 25.22%, and 27.13% under T3 treatment; decreased by 53.23%, 28.46%, 34.33%, and 36.94% under T4 treatment; and decreased by 142.77%, 76.85%, 53.66%, and 53.03% under T5 treatment, respectively (Figure 3A). Additionally, the forage yield of dry weight in each growth period decreased by 13.06%, 1.92%, 13.59%, and 14.90% under T2 treatment compared with T1, respectively; decreased by 21.06%, 16.27%, 17.48%, and 21.90% under T3 treatment; decreased by 45.74%, 31.79%, 25.32%, and 30.18% under T4 treatment; and decreased by 182.41%, 110.94%, 76.12%, and 58.93% under T5 treatment, respectively (Figure 3B).

### 2.2. Photosynthetic Pigment and Assimilates

#### 2.2.1. Chlorophyll Content

**Figure 4:**
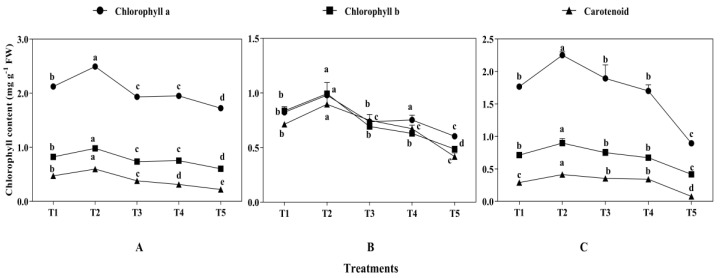
The effect of mixed cropping ratios of oat and alfalfa on chlorophyll content in saline soil. Different letters indicate significant differences between different treatments at the same growth stage at the *p* < 0.05 level. ((**A**), 60 DAS; (**B**), 89 DAS; (**C**), 121 DAS).

Leaf chlorophyll contents were significantly affected by mixed cropping treatment (*p* < 0.05, Figure 4). The chlorophyll contents were increased first and then declined with decreased oats and increased alfalfa sowing rate. The highest contents showed under the T2 treatment and the lowest under the T5 treatment at all growth periods. At 60 DAS, T2 produced 17.45% higher chlorophyll a, 19.51% chlorophyll b, and 21.67% carotenoids than that under T1, and 44.77%, 61.66%, and 63.33% higher chlorophyll a, chlorophyll b, and carotenoids than that under T5, respectively (Figure 4A). At 89 DAS, the content of chlorophyll a under T2 was 14.85% higher than that of under T1 treatment and 116.82% higher than that under T5. Similarly, the contents of chlorophyll b and carotenoids under T2 were 17.86% and 25.64%, 102.04% and 66.67% higher than that under T1 and T5, respectively (Figure 4B). At 121 DAS, the contents of chlorophyll a, chlorophyll b, and carotenoids under T2 were 27.12%, 25.35%, and 29.67% higher than those under T1, and 152.81%, 111.90%, and 80.48% higher than those under T5, respectively (Figure 4C).

#### 2.2.2. Soluble Sugar, Sucrose and Starch

**Figure 5:**
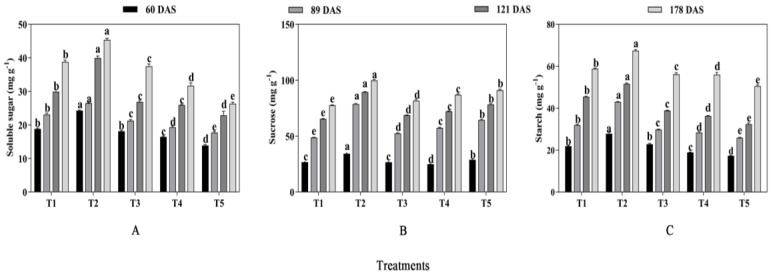
Effects of mixed cropping ratios of oat and alfalfa on soluble sugar, sucrose, and starch contents in saline soil. Different letters indicate significant differences between different treatments at the same growth stage at the *p* < 0.05 level. ((**A**), soluble sugar content; (**B**), sucrose content; (**C**), starch content).

As shown in Figure 5, the contents of soluble sugar, sucrose, and starch were significantly affected by mixed cropping treatment (*p* < 0.05). The soluble sugar and starch showed a trend of increasing firstly and then declining with decreased oats and increased alfalfa sowing rate at each growth period. Consistently, the maximum contents were produced under the T2 treatment, followed by the T1 treatment, while the minimum contents were all shown under the T5 treatment at all periods (Figure 5A,C). At 60 DAS, the soluble sugar showed 29.26% and 74.82% higher content, and starch showed 26.94% and 61.76% higher content under T2 than that under T1 and T5, respectively. At 89 DAS, T2 treatment increased soluble sugar by 14.72% and 49.72% and starch content by 34.80% and 66.02% compared with T1 and T5 treatment, respectively. At 121 DAS, the contents of soluble sugar and starch under T2 treatment were 33.89% and 13.66% higher than that under T1 treatment and 74.24% and 59.26% higher than that under T5, respectively. Similarly, at 178 DAS, T2 treatment increased the contents of soluble sugar and starch by 17.01% and 14.63% compared with T1 and by 72.62% and 33.47% compared with T5, respectively.

However, the content of sucrose showed the highest value under T2, followed by T5, while the lowest value was generated under T1 treatment (Figure 5B). In general, the T2 treatment produced 28.46% and 18.69% higher sucrose content than T1 and T5 at 60 DAS, 61.40% and 22.05% at 89 DAS, 36.54% and 13.90% at 121 DAS, and 28.61% and 9.55% at 178 DAS, respectively. Overall, the contents of soluble sugar, sucrose, and starch showed a gradual decrease with decreased oats and increased alfalfa sowing rate (Figure 5).

### 2.3. Membrane Lipid Peroxidation and Osmoregulation Substances

**Figure 6:**
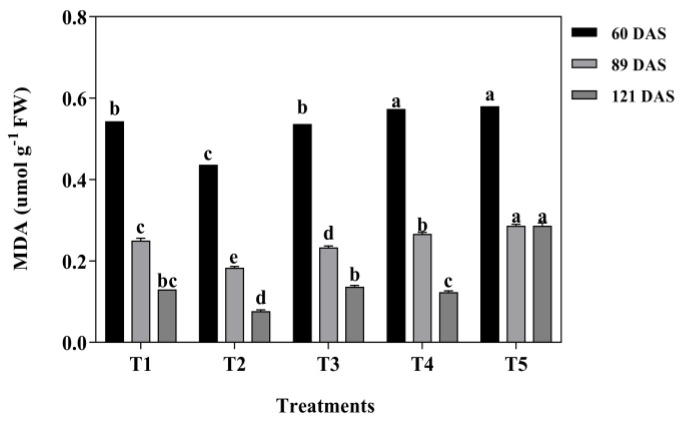
Effects of mixed cropping ratios of oat and alfalfa on MDA content in saline soil. Different letters indicate significant differences between different treatments at the same growth stage at the *p* < 0.05 level.

**Figure 7:**
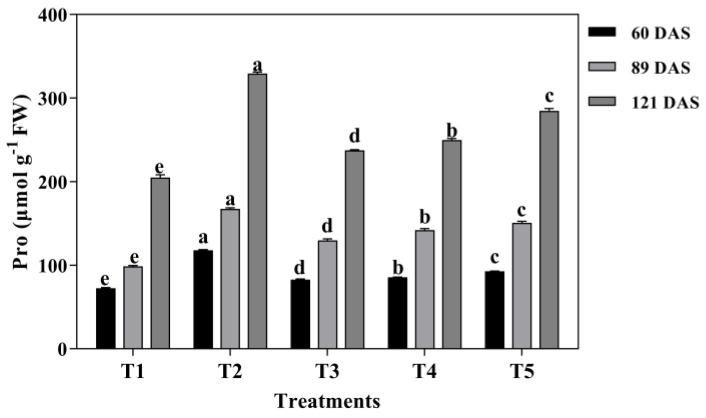
Effects of mixed cropping ratios of oat and alfalfa on Proline (Pro) content in saline soil. Different letters indicate significant differences between different treatments at the same growth stage at the *p* < 0.05 level.

According to Figure 6 and Figure 7, it is evident that the membrane lipid peroxidation of MDA and osmoregulation substances of Pro were significantly affected by mixed cropping treatment (*p* < 0.05).

However, the variation tendency was the opposite between MDA and Pro under each treatment and at all growth periods. The content of MDA showed a decrease first and then increased with decreased oats and increased alfalfa sowing rate at each growth period. Both the lowest contents of MDA and the highest contents of Pro were generated under T2 treatment at each growth period. Compared with T1 and T5, the contents of MDA decreased by 18.52% and 31.82% under T2 treatment at 60 DAS, by 38.89% and 61.11% at 89 DAS, by 62.50% and 262.5% at 121 DAS, respectively (Figure 6). Nevertheless, the content of Pro showed an increased tendency with decreased oats and increased alfalfa sowing rate. Contrary to MDA, T2 treatment showed 62.26% and 27.08% higher content of Pro than that of T1 and T5 at 60 DAS, 69.68% and 10.94% at 89 DAS, and 60.74% and 15.67% at 121 DAS, respectively (Figure 7).

### 2.4. Antioxidant Enzyme Activity

**Figure 8:**
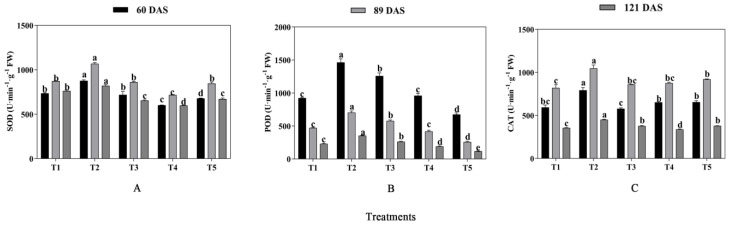
Effects of mixed cropping ratios of oat and alfalfa on SOD, POD, and CAT activity in saline soil. Different letters indicate significant differences between different treatments at the same growth stage at the *p* < 0.05 level. (**A**), SOD; (**B**), POD; (**C**), CAT.

It can be seen from Figure 8 that the activities of SOD, POD, and CAT were significantly affected by mixed cropping treatment (*p* < 0.05). On the whole, the activities of antioxidant enzymes were all increased from T1 to T2 and decreased from T2 to T5 with decreased oats and increased alfalfa sowing rate at each growth period. The highest activities were concordant and showed under T2 treatment (Figure 8). At 60 DAS, T2 generated 19.23% and 29.22% higher SOD activities, 58.56% and 116.98% higher POD activities, and 33.51% and 20.89% higher CAT activities than T1 and T5 treatments, respectively. At 89 DAS, the activities of SOD, POD, and CAT under T2 treatment were 22.32%, 48.95%, and 27.87% higher than that under T1, and 26.19%, 171.96%, and 13.82% higher than that under T5 treatments, respectively. Similarly, T2 treatment increased by 7.72% and 22.29% for SOD, by 53.26% and 199.75% for POD, and by 26.78% and 19.19% for CAT compared with T1 and T5 at 121 DAS, respectively (Figure 8).

### 2.5. Nutrient Accumulation

**Figure 9:**
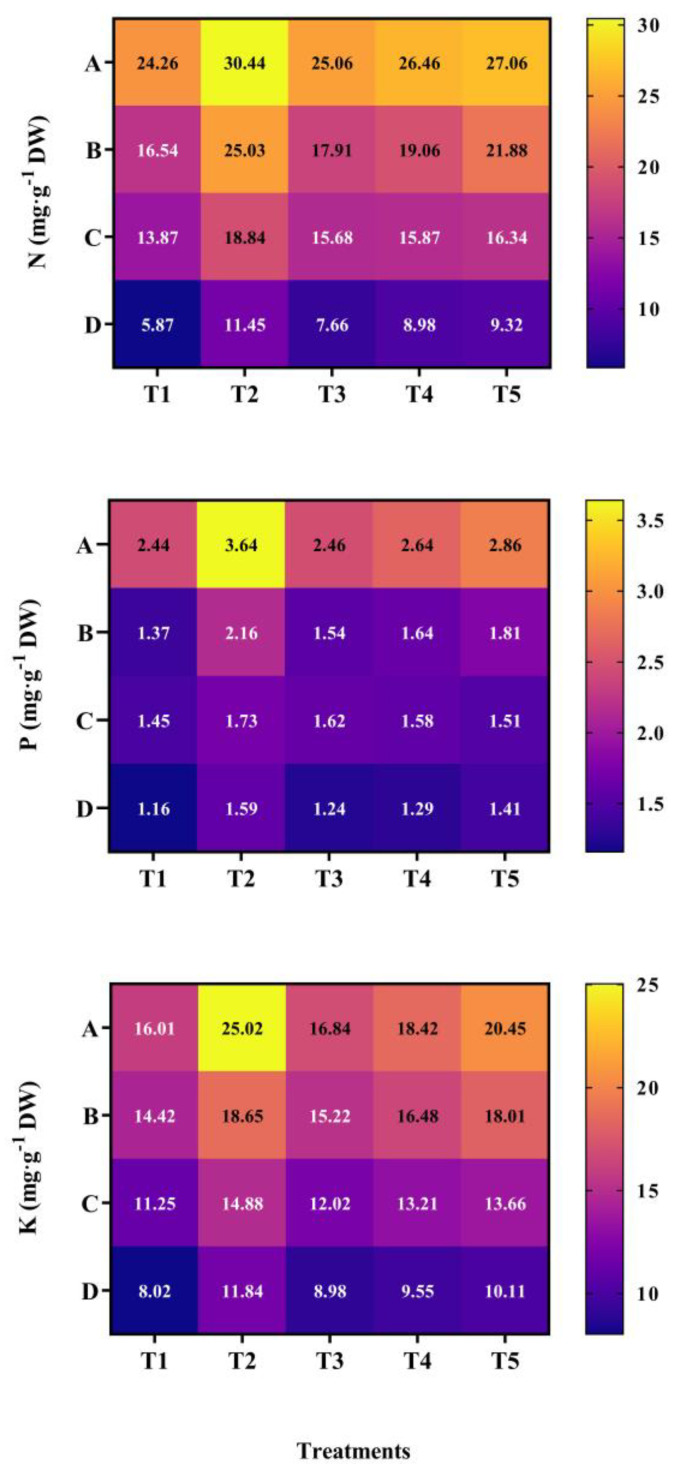
Effect of mixed cropping ratios of oat and alfalfa on nitrogen, phosphorus, and potassium content in saline soil. A, 60 DAS; B, 89 DAS; C, 121 DAS; D, 189 DAS.

It was evident that the accumulation of nitrogen, phosphorus, and potassium was significantly affected by mixed cropping treatment (*p* < 0.05, Figure 9). At each growth period, the content of N, P, and K all increased with decreased oats and increased alfalfa sowing rate (compared with T1), and the highest contents were all shown under T2 treatment. At 60 DAS, the T2 treatment showed 26.96% and 12.31% higher N content, 46.91% and 25.26% P content, and 57.51% and 24.61% K content than the T1 and T5 treatments, respectively. At 89 DAS, the contents of N, P, and K under T2 treatment increased by 51.34%, 57.66%, and 30.38% compared with T1, and 13.36%, 18.03%, and 4.29% compared with T5. In addition, at 121 DAS and 189 DAS, the N content of N2 increased by 33.10% and 100.17% compared with T1, by 13.20% and 22.53% compared with T5. The P content under T2 was 18.62% and 37.07% higher than T1 and 14.67% and 12.77% higher than T5, respectively. The K content was 32.35% and 44.64% higher than T1 and 8.26% and 16.02% higher than T5, respectively (Figure 9).

### 2.6. Forage Quality

**Figure 10:**
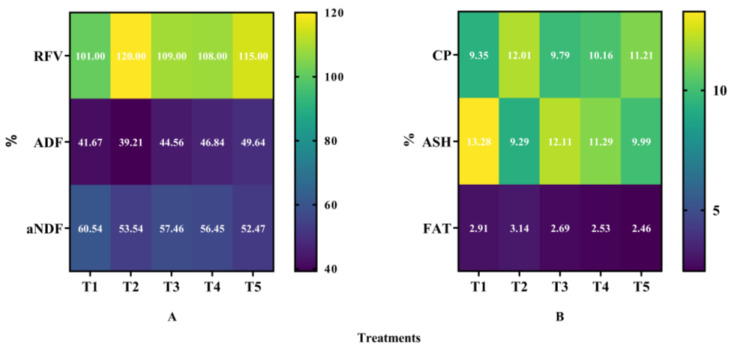
Effect of mixed cropping ratios of oat and alfalfa on forage quality in saline soil. (**A**): RFV, relative feeding value; ADF, acid detergent fiber; aNDF, neutral detergent fiber. (**B**): CP, crude protein; ASH, crude ash; FAT, crude fat.

It clearly showed that the mixed cropping ratio of oats and alfalfa significantly affected forage quality (*p* < 0.05, Figure 10). Among them, the relative feeding value (RFV) and crude protein (CP) significantly increased with decreased oats and increased alfalfa sowing rate. The highest contents were performed under the T2 treatment, which produced 17.70% higher RFV and 28.45% higher CP content than T1, respectively. On the contrary, the contents of acid detergent fiber (aNDF) and crude ash (ASH) significantly decreased with decreased oats and increased alfalfa sowing rate, and the lowest values were generated under T2 treatment. Compared with T1, the contents of aNDF and ASH decreased by 11.56% and 30.05% under T2 treatment, respectively. However, the content of neutral detergent fiber (ADF) decreased first and then increased with decreased oats and increased alfalfa sowing rate. The lowest content of ADF was under T2 treatment, which decreased by 5.90% compared to T1. Furthermore, the highest content of ADF showed under the T5 treatment, which was 19.13% higher than that under the T1 treatment (Figure 10A). Opposite to ADF, the content of crude fat (FAT) was increased firstly and then decreased with decreased oats and increased alfalfa sowing rate. The content of FAT increased by 7.53% under T2 but decreased by 28.16% under T5 compared with T1, respectively (Figure 10B).

## 3. Discussion

### 3.1. Effect of Mixed Cropping Treatment on Growth Traits of Oat and Alfalfa

Salinity is a major abiotic stress, limiting crop growth and reducing grain yield [18]. At present, screening and breeding of salt-tolerant crop varieties and the development of salt-tolerant-promoting cultivation techniques are two major approaches to improving and utilizing saline soils [19]. In the present study, the results demonstrated that mixed cropping oats with alfalfa in an appropriate ratio can significantly improve plant growth, biomass yield, and forage quality in saline soil. It has illustrated that the mixed cropping system improves forage productivity and is attributed to optimizing the community’s spatial distribution and resource allocation, such as nutrients and sunlight [20]. The results of this study indicate that different mixing ratios of oats and alfalfa can significantly affect plant growth, biomass yield, and forage quality in saline soils. Chen et al. suggested that an optimal sowing ratio can enhance the spatial distribution of communities and effectively utilize resources such as soil nutrients and sunlight. These can lead to a dynamic change in plant height, growth of stems and leaves, and the ability to tiller and branch, which are important factors reflecting forage growth and development, closely related to yield [21,22]. Furthermore, the composite populations formed by intercropped species can enhance light transmission, reduce energy waste, facilitate plant growth and development, and promote biomass accumulation within the communities [23,24]. Compared to the T1 treatment of sole oats cultivation, the plant height under T2 treatment increased while it decreased under T3 and T4 treatments in the mixed cropping conditions. Biomass yield also decreased with the reduction in oat seeding rate. In comparison to the T5 treatment of sole alfalfa cultivation, under mixed cropping conditions, both plant height and biomass yield for treatments T2 to T4 continuously decreased with the declining oat seeding rate. These results suggest that mixed cropping may have a certain promoting effect on oat growth but some negative effects on leguminous forage. This conclusion is supported by the previous study that leguminous forage plants (alfalfa) showed a weaker competitive ability in plant growth and relative growth rate than oats plants in the mixed cropping system under adverse conditions [25].

### 3.2. Effect of Mixed Cropping Treatment on Physiological Characteristics of Oat and Alfalfa

Saline soils have high levels of soluble salts and exchangeable sodium (Na^+^) and chlorine (Cl^−^), which usually pose a series of stresses, including osmotic stress, ionic imbalances, and secondary stresses like nutritional imbalances and oxidative stress and damage to plants [26]. In the present study, higher content of MAD, lower content of chlorophyll, soluble sugar, proline, starch, N, P, and K, and lower activity of SOD, POD, and CAT were shown under T1 or T5 (oat monocropping or alfalfa monocropping) than other treatments (mixed cropping treatment) in saline soil. These results demonstrated that monoculture plants accumulate excess Na^+^ in saline soil and exert ionic toxicity effects that reduce uptake of other nutrition ions and injure cell membranes, which leads to the generation of reactive oxygen species (ROS) in all cell compartments to damage DNA, proteins, pigments, and membranes [3]. These processes could decrease biomass accumulation and allocation, which could further decrease plant height, RGR, biomass yield, and forage quality, as shown in this study. On the other hand, it illustrated that the mixed cropping treatment can prominently regulate the physiological metabolism of forage plants under salt conditions. As shown in this study, more osmotic adjustment substances of soluble proline and higher antioxidant enzyme activity of SOD, POD, and CAT were generated under mixed cropping treatments, especially under T2 (oat 75%+alfalfa 25%), which could effectively alleviate the damage of ROS (reactive oxygen species, including superoxide (O_2_^−^), hydroxyl radicals (OH), hydrogen peroxide (H_2_O_2_), and singlet oxygen (O_2_)) that generate under salt stress on DNA, proteins, pigments, and membranes, resulting in more assimilate accumulation (soluble sugar, starch, sucrose) and biomass yield production.

Chlorophyll is an important parameter for plants, which directly affects photosynthesis and assimilate accumulation [27]. Our results are consistent with the previous study that the chlorophyll content of oats mixed with alfalfa is 5% to 37% higher compared to sole alfalfa seeding [28,29]. Starch and sucrose are important products of photosynthesis, which is closely related to chlorophyll [30]. As shown in the present study, the variation tendency of starch, sucrose, and chlorophyll was similar under mixed cropping treatment during each growth period. N and P are the essential elements required for plant growth, but deficiencies of N and P are common in saline soils. In saline soil, forage plants mainly experience osmotic stress, and more Na^+^ accumulates into the plant tissue, which limits the nutrient elements of N, P, and K acquisition. In the present study, mixed cropping plants uptake more nutrition than monocropping plants because the optimized canopy structure by mixed cropping treatment can enhance root development and absorbing ability. Additionally, they can improve the nitrogen-fixing capacity of alfalfa, which is beneficial for mixed cropping oat plants [31].

### 3.3. Effect of Mixed Sowing on Forage Quality of Oat and Alfalfa

Crude protein, neutral detergent fiber (NDF), and acid detergent fiber (ADF) are important indicators for evaluating the nutritional quality of forage, and the relative feed value (RFV) is currently the most widely used index for assessing the quality of roughage [32]. Usually, low content of crude protein and high levels of NDF and ADF reflect low digestibility and poor forage quality, leading to a lower RFV. The current study indicates that oat mixed cropping with alfalfa significantly improved the nutritional quality and RFV compared to oat or alfalfa monocropping. Similar results were found in other crops. It has been shown that mixed cropping legumes and grasslands can reduce the content of ADF and NDF, improve grass quality, and provide high-quality forage for livestock [33]. Mixed cropping sorghum with cowpea increased the crude protein content and reduced the levels of NDF and ADF, thereby enhancing the RFV and improving the quality of grass [34]. Lazarev et al. [35] demonstrated that intercropping sorghum with alfalfa significantly increased the crude protein content and RFV of silage. Overall, the nutritional quality of mixed forage was superior under mixed cropping treatment rather than monocropping cultivation. Moreover, the nutritional quality of mixed cropping forage depends largely on the mixed species, mixed cropping ratio, and sowing time [36,37,38].

## 4. Materials and Methods

### 4.1. Experimental Design and Site

The field experiment was conducted on the Dafeng Coastal Forest Farm of Dafeng, in Yancheng City (33°20′N, 120°47′E), Jiangsu Province, China, in the growing seasons of 2019 and 2020. This region belongs to the transitional zone from subtropical to warm and humid climate, with four distinct seasons, moderate temperatures with an average annual temperature of 14.1 °C, abundant rainfall with an annual precipitation of 1042.2 mm, a frost-free period of 213 days, and an annual sunshine duration of 2238.9 h. The soil in the experimental field had the texture of clay loam with organic matter content 21.43 g ha^−1^, total N 0.81 g ha^−1^, available P 1.51 mg ha^−1^, available K 266.00 mg ha^−1^, pH 8.4, salt content 1.86 g kg^−1^, and average electrical conductivity (EC) of 10.87 mS cm^−1^ in the 0–20 cm soil layer.

The oat variety of Canadian Highlander and the alfalfa variety of WL525HQ were used in this study. The seeds were harvested in the previous year; high-quality seeds were robust and full, without damage or insect infestation, and stored under good conditions. Five mixed cropping ratios were arranged (Table 1). Oat and alfalfa were sowing in a drill with a row space of 30 cm. All the seeds were sown on November 15 each year. Nitrogen fertilizer was applied as urea (46% N) at a rate of 225.0 kg N ha^−1^, while phosphorus fertilizer was applied as calcium superphosphate (12% P_2_O_5_), equivalent to a rate of 75.0 kg ha^−1^ P_2_O_5_. All the fertilizers were equally separated into two parts and applied as basal fertilizer before sowing and topdressing fertilizer at the seedling stage (50 DAS, day after seeding), respectively. The experiment was arranged in a randomized complete block design with three replicates, a total of 15 plots, and each plot 30 m^2^ (3 m × 10 m). Other field practices were used in conformity with local recommendations to avoid yield losses.

### 4.2. Observations and Measurements

#### 4.2.1. Growth Traits and Forage Yield

At 60, 89, 121, and 178 days after sowing (DAS) (corresponding to the seedling stage, tillering stage, jointing stage, and milk ripening stages for oats, and the seedling stage, bud stage, blooming stage, and seed-bearing stages for alfalfa, respectively), about 1 m^2^ (1.0 m × 1.0 m) of forage plants was harvested randomly in each plot. After measurements of plant height and fresh weight, the sampled plants were oven-dried at 80 °C to constant weight for dry biomass determination (Yiheng Oven-DHG-9920A, Shanghai Yi Heng Instrument Co., Ltd., Shanghai, China). Forage yield was expressed as fresh weight (fresh forage yield) and dry weight (dry forage yield) at each sampling period. After that, the relative growth rate (RGR) was calculated using the following formula: RGR (kg ha^−1^ d^−1^) = (W2 − W1)/(t1 − t2), where W1 and W2 were the dry biomass measured at S1 and S2 stages, respectively [19]. Then, the dried plants were ground into powder, sieved through a 100-mesh screen, and stored in sealed bags to determine N, P, K, and other parameters.

#### 4.2.2. Physiological Parameters

At each growth stage, the upper expanded leaves from 5 plants in each plot were sampled and immersed in liquid nitrogen immediately, after that stored in an ultra-low temperature refrigerator (−80 °C) for physiological assays.

(1) Photosynthetic pigment and assimilates accumulation

Chlorophyll content: About 0.5 g of leaf samples were extracted in 80% (*v*/*v*) aqueous acetone overnight. The optical density was measured at 663, 652, and 645 nm using a spectrophotometer (Beckman Coulter, Inc., Brea, CA, USA). The contents of chlorophyll a and chlorophyll b were calculated by the following equations:

Chl a (mg g^−1^ FW) = (12.21 × Abs663 − 2.81 × Abs645) × V/(1000 × W)

Chl b (mg g^−1^ FW) = (20.13 × Abs645 − 5.03 × Abs663) × V/(1000 × W)

where Abs is the absorbance, V is the final volume of the extract (mL), and W is the weight of the leaf sample (g) used [20].

Soluble sugar and starch content: A 0.1 g powder sample was extracted with 80% ethanol in an 80 °C water bath for 30 min. After centrifugation, the supernatant was transferred to a 100-mL volumetric flask. The extract was repeated three times. A total of 2 mL distilled water was added to the residue and incubated in a 100 °C water bath for 15 min. After cooling, 2 mL of 9.2 mol L^−1^ perchloric acid (HClO_4_) was added, and the mixture was incubated in an ice bath for 15 min before extraction. After centrifugation, the supernatant was transferred to a 100 mL volumetric flask to assay soluble sugar. Then, 2 mL of 4.6 mol L^−1^ HClO_4_ was added to the residue for extraction, and the supernatants from two rounds of centrifugation were combined in the same volumetric flask and brought to 100 mL with distilled water. This mixture was used to determine the starch content. The contents of soluble sugars and starch were measured at 620 nm on a microplate reader (Nano Quant, infinite M200, Tecan, Switzerland) using a colorimetric method with the anthrone reagent [38].

Sucrose content: 0.1 g of the sample was weighed and placed in a centrifuge tube, followed by the addition of 6 mL of 80% ethanol and incubation in a water bath at 80 °C for 30 min. After cooling, the mixture was centrifuged for 15 min and filtered, and the process was repeated three times. The filtrates were combined into a solution, constituting the extract. In total, 1 mL of the extract, 1 mL of ultrapure water, and 4 mL of anthrone solution were mixed and boiled in a water bath for 15 min, followed by cooling and measurement of absorbance at 480 nm [39].

(2) Membrane lipid peroxidation and osmoregulation substances

Malonaldehyde (MDA) content: About 0.5 g of fresh leaf was ground in 0.1% trichloroacetic acid (TCA), then mixed and centrifuged at 12,000× *g* for 15 min to prepare for the MDA extraction. After that, 1 mL supernatant with 4 mL 0.5% thiobarbituric acid (containing 20% trichloroacetic acid) was heated at 95 °C for 15 min and then centrifuged at 10,000× *g* for 15 min. Then, the sample was recorded for absorption at 600, 532, and 450 nm, and MDA content was calculated [40].

Proline (Pro) content: About 0.5 g fresh leaf was ground in an 80% anhydrous ethanol solution to prepare proline extraction, and then 0.4 g artificial zeolite and 0.2 g activated carbon were added and fully oscillated for 5 min to remove the interference of other amino acids and filtrate for backup. After that, 2 mL of the extract was placed in a test tube, followed by adding 2 mL glacial acetic acid and 2 mL of newly prepared acid triketone solution (2.5 g ninhydrin dissolved in 60 mL glacial acetic acid and 40 mL 6 mol L^−1^ phosphoric acid). The absorbance was recorded at 520 nm after heating in a boiling water bath for 15 min. The content of free proline in each sample was determined using the standard curve of preparation of analytical grade proline [41].

(3) Antioxidant enzymes

Peroxidase (POD) activity: About 0.1 g of frozen leaf was ground in 3 mL of 0.1 mol L^−1^ phosphate buffer (pH 7.0) to extract POD. After that, the extraction was centrifuged at 18,000× *g* at 4 °C for 15 min. The supernatant was used as the enzyme source. The oxidized o-diphenylamine was determined at 430 nm. Phosphate buffer (0.1 mol L^−1^, pH 6.5) was placed in colorimetric dishes containing enzyme extract. Then, 0.2 mL 0.2 mol L^−1^ H_2_O_2_ was added and mixed, and the absorbance per min was recorded. The POD activity unit is expressed as the rate of increase in absorbance per min [42].

Catalase (CAT) activity: About 0.1 g of frozen leaf was homogenized in 5 mL assay mixtures, which contained 2.9 mL substrate solution (30% hydrogen peroxide in 50 mmol L^−1^ potassium phosphate buffer) and 0.1 mL of enzyme extraction. The decomposition of H_2_O_2_ was stopped by adding 2 mL potassium dichromate (5%) to the mixed solution. The absorbency was measured immediately at 240 nm and read every 30 s for 2 min to calculate the CAT enzyme activity [43].

Superoxide dismutase (SOD) activity: 1.0 g of the frozen leaf was ground with 3 mL of 0.05 mol L^−1^ PBS buffer (pH = 7.8) and a small amount of quartz sand in an ice bath, then transferred into a 5 mL centrifuge tube and centrifuged at 10,000 r min^−1^ for 15 min at 4 °C, and the supernatant was extracted as the enzyme solution. The SOD reaction solution (5 mL 100 mmol L^−1^ potassium phosphate buffer (pH 7.8), which contains 0.1 mmol L^−1^ EDTA (ethylenediamine tetraacetic acid disodium salt), 0.1% Triton X-100, and 2% polyvinyl pyrrolidone), and 1 mL enzyme solution were taken in 10 mL centrifuge tubes and immediately subjected to fluorescent tube light at 4000 LX, and the reaction was terminated by stopping the light and shading the light after 15 min. The absorbency was measured at 560 nm wavelength colorimetric. One unit of SOD activity is expressed as the amount of enzyme required to cause 50% inhibition of epinephrine oxidation [19].

(4) Nutrient accumulation—nitrogen, phosphorus, and potassium

Nitrogen content: 0.5 g of dried sample was weighed and placed in a glass test tube, followed by the addition of concentrated sulfuric acid and catalysts (prepared with copper sulfate and potassium sulfate). The reaction was carried out at 420 °C for 1.5 h. After the reaction was completed, the tube was cooled in a ventilated area until all harmful gases had completely evaporated. After cooling, the ammonium nitrogen content (mg N/g) could be determined using a Kjeldahl nitrogen analyzer (K1160, Hanon Instrument Co., LTD, Jinan, China) [42].

Phosphorus content: in an acidic solution, orthophosphoric acid in the digestion solution reacted with ammonium metavanadate and ammonium molybdate to form a yellow complex phosphomolybdate, which was left at room temperature above 15 °C for 30 min. The absorbance was measured at a wavelength of 700 nm using a spectrophotometer, and the content was calculated [43].

Potassium content: potassium content was determined using atomic absorption spectrophotometry at a resonance line of 766.5 nm [43].

(5) Forage quality—crude fat, crude protein, and crude fiber

The feed quality of these plants was also determined, including the relative feed value, the acidic washed fiber, the neutral washed fiber, crude protein, crude fat, and crude ash [44].

### 4.3. Statistical Analysis

Analysis of variance (ANOVA) was performed using Statistix 9 (Analytical Software, Tallahassee, FL, USA), and mean values were compared based on the least significant difference (LSD) at the 0.05 probability level. Figures were generated using SigmaPlot 10.0 (SPSS Inc., Point Richmond, CA, USA). The significant difference letters in figures were marked according to the result of multiple comparisons (*p* < 0.05) by Statistix 9 (Analytical Software, FL, USA). All the parameters are shown as the average values of the 2-year experiments because the tendency of each parameter was similar in each year, and there was no significant difference between the two years.

## 5. Conclusions

Compared with monoculture, mixed cropping of oats and alfalfa could significantly improve plant growth, forage yield, and forage quality in saline soil. Among the treatments, the mixed cropping treatment of T2 showed the best performance in improving both biomass yield and forage quality by enhanced antioxidant enzyme activity, osmotic regulatory substances, and nutrient uptake and utilization. Therefore, this study indicates that 75% (135 kg ha^−1^) oat mixed cropping with 25% (7.5 kg ha^−1^) alfalfa can be recommended as a salt-tolerant cultivation technique for forage high-yield and high-quality production in moderately saline soil.

## Figures and Tables

**Table 1:** The seeding rate of oats and alfalfa in the mixed cropping system.

	Oat Seeding Rate (kg ha^−1^)	Alfalfa Seeding Rate (kg ha^−1^)	Mixed Seeding Ratio
T1	180.0	0	100% oat + 0% alfalfa
T2	135.0	7.5	75% oat + 25% alfalfa
T3	90.0	15.0	50% oat + 50% alfalfa
T4	45.0	22.5	25% oat + 75% alfalfa
T5	0	30.0	0% oat + 100% alfalfa

## Data Availability

Data are contained within the article.
